# Impacts of infectious diseases on movement metrics in a large carnivore: Highly pathogenic avian influenza, leptospirosis, and pumas

**DOI:** 10.1016/j.isci.2025.113810

**Published:** 2025-10-17

**Authors:** Fernando Nájera, Stella Uiterwaal, Dave K. Garcelon, John F. Randolph, T. Winston Vickers, Phillip Johnston, Thomas R. Stephenson, L. Mark Elbroch, Jason V. Lombardi, Deana L. Clifford

**Affiliations:** 1 Karen C. Drayer Wildlife Health Center, California Carnivores Program, School of Veterinary Medicine, University of California, Davis, One Shields Avenue, Davis, CA 95616, USA; 2 Smithsonian Conservation Biology Institute, 1500 Remount Road, Front Royal, VA 22630, USA; 3 Institute for Wildlife Studies, P.O. Box 1104, Arcata, CA 95518, USA; 4 Mountain Lion Conservation Program, Wildlife Health Laboratory, California Department of Fish and Wildlife, 1701 Nimbus Road, Rancho Cordova, CA 95670, USA; 5 Sierra Nevada Bighorn Recovery Program, California Department of Fish and Wildlife, 787 North Main Street, Suite 220, Bishop, CA 93514, USA; 6 Panthera, 104 West 40^th^ Street, 5^th^ Floor, New York, NY 10018, USA

**Keywords:** Wildlife epidemiology, Ecology

## Abstract

Understanding how behavior and movement are affected during the clinical stages of infectious disease in hosts provides stronger insight into activity patterns. We identified changes in movement and behavioral metrics in seven satellite-collared pumas (*Puma concolor couguar*) that succumbed to *Leptospira* or highly pathogenic avian influenza (HPAI). We used GPS data from pumas in California and Washington to estimate disease onset dates and investigated movement and behavioral (kill site, bed site, and slow local movement) changes before and after onset. Six pumas exhibited changes in movement metrics, along with longer bouts of those behaviors. For the seventh, consistently low movement precluded comparisons to normal behavior. Based on the onset dates, HPAI infection resulted in a more acute death than *Leptospira* infection. Our results highlight the potential of movement and behavioral metrics to study infectious disease effects on pumas and other carnivores, contributing to epidemiological and clinical features of these diseases in carnivores.

## Highlights

• GPS collar data reveal disease-related shifts in free-living pumas' movement and behavior
• Movement data unfold the hidden stages of infectious diseases in pumas
• Step length reductions reliably signal the disease onset of HPAI and leptospirosis
• Infected pumas showed extended bouts of kill, bed, and slow behaviors

## Graphical abstract

**Figure:**
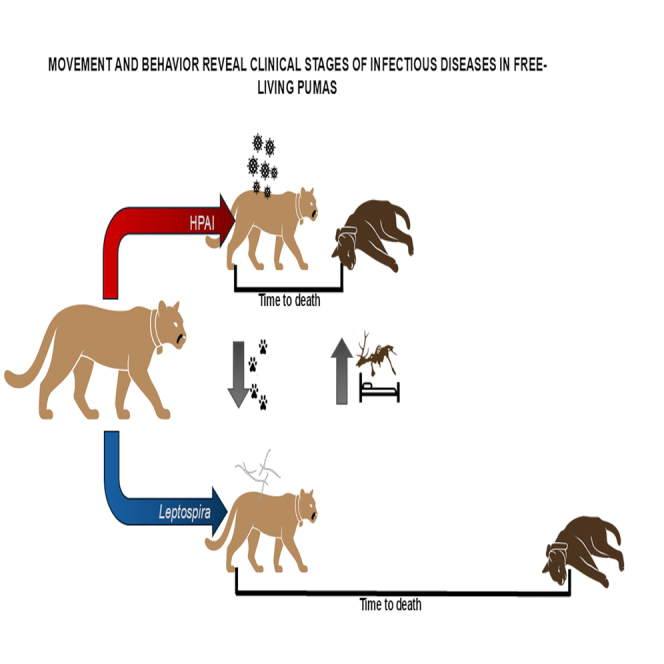

## Introduction

Infectious diseases are a growing threat to many wildlife populations, but especially those already vulnerable due to small size or isolation.^1^^,^^2^^,^^3^^,^^4^ Large carnivore population declines, for example, have been linked to habitat loss, habitat fragmentation, habitat transformation, and direct exploitation.^3^^,^^5^ Many small carnivore populations are now additionally threatened by disease (e.g., devil facial tumor disease and Tasmanian devils (*Sarcophilus harrissi*)^6^; canine distemper, rabies, and island fox (*Urocyon littoralis*)^7^^,^^8^; canine distemper, rabies, and Ethiopian wolf (*Canis simensis*).^9^ This may also be the case for some puma (*Puma concolor couguar*) populations in Florida, California, and Washington, where low genetic diversity, high inbreeding, and population fragmentation have placed some regional populations at risk of extirpation^10^^,^^11^^,^^12^^,^^13^^,^^14^^,^^15^^,^^16^ and for whom disease risk may accelerate population decline. Feline leukemia virus (FeLV) and, more recently, feline leukomyelopathy, are of high concern in the small, isolated Florida panther population.^4^^,^^17^

Disease may also impact fitness by altering the behavior of infected individuals, affecting the ability to hunt or defend territory. Understanding the behavior of infected individuals, thus, contributes to our knowledge of disease epidemiology and ultimately informs the development of effective disease management measures.^18^^,^^19^ While the behaviors of free-ranging species can be difficult to observe directly, satellite telemetry can provide insight into their activity patterns. Movement metrics, as well as some basic behavioral states, can be detected from animal tracking data and have the potential to provide insight into the impacts of disease on wild animals. For example, coyotes (*Canis latrans*) infested with *Sarcoptes scabiei* have larger monthly home ranges^20^ while wolves (*Canis lupus*) suffering from sarcoptic mange show a decrease in daily movements.^21^ Animal tracking data may also inform transmission risk; widely roaming infectious individuals could disproportionately increase disease spread (e.g., rabies and raccoons, *Procyon lotor*),^22^^,^^23^ while high immobility behavior displayed by highly pathogenic avian influenza-infected griffon vultures (*Gyps fulvus*) could reduce the risks of transmission.^24^

Pumas are exposed to a wide range of infectious diseases that, combined with ongoing habitat loss and modification, deserve attention due to their potential negative impact on population viability.^4^^,^^25^ Pumas have shown exposure to feline pathogens (e.g., feline panleukopenia virus, feline calicivirus, feline coronavirus, feline herpesvirus, feline leukemia virus, feline immunodeficiency virus), canine pathogens (e.g., heartworm, canine distemper virus), zoonotic vector-borne bacterial pathogens (e.g., *Yersinia pestis*, *Francisella tularensis*, *Bartonella henselae*, *Borrelia burgdorferi*, and *Anaplasma phagocytophilum),* and protozoal pathogens (e.g., *Toxoplasma gondii*).^25^^,^^26^^,^^27^^,^^28^

Through case examples obtained from satellite-collared pumas, this study aims to identify movement and behavior metrics that may be impacted by infectious diseases, focusing on leptospirosis and highly pathogenic avian influenza (HPAI). Leptospirosis is a potentially fatal zoonotic disease caused by spirochetes in the genus *Leptospira,* for which the course of infection and clinical signs can range from practically inapparent to severe and fatal, depending on the dose of organisms received and the susceptibility of the species.^29^^,^^30^ Research on leptospirosis has shown that it is a common, widespread, and chronic infection in California’s pumas and may have clinical impacts on the renal and overall health of individuals.^31^ Although in California, *Leptospira* infection appears to be associated in a few instances with probable clinically significant nephritis,^31^ it has the potential to cause mortality in the species.^32^

Highly pathogenic avian influenza virus (H5N1) is a recent panzootic that has demonstrated abilities to infect non-avian species.^33^^,^^34^^,^^35^ Within Mammalia, the order Carnivora may be especially at risk of infection via the ingestion of infected prey (i.e., birds).^36^ Environmental contamination could also be a source of transmission, and recent viral mutations may increase the probability of mammal-to-mammal transmission.^34^ HPAI in felids is usually fatal,^37^^,^^38^ although subclinical infections have been reported.^39^^,^^40^^,^^41^ In the USA, the first reported case of HPAI in pumas occurred in Nebraska in January 2023.^42^ To date, California and Washington have detected fatal HPAI infection in 12 pumas, 35% (12/34) of the puma cases detected in the country.^42^

Domestic cats (*Felis sylvestris catus*) infected with HPAI H5N1 can present with subclinical infections or develop rapidly progressive, severe disease^39^^,^^43^^,^^44^ with clinical signs ranging from high fever from day 1 post-infection and, by day 2 and depending on the systems affected, lethargy, protrusion of the nictitating membranes, conjunctivitis, dyspnea, serosanguinous nasal discharge, icterus, convulsions, ataxia or other neurological/gastrointestinal signs, and a high fatality rate.^45^ There is a paucity of information regarding subclinical infections of HPAI in wild felids. Clinical signalment in wild felids has only been reported in bobcats (*Lynx rufus*) and tigers (*Panthera tigris tigris*) and included lethargy, dyspnea, neurologic abnormalities such as tremors, and lack of fear (bobcats)^46^; high fever, respiratory distress, serosanguinous nasal discharge, and neurologic signs (tigers).^47^

We investigated four movement metrics (step lengths, turn angles, daily movement distances, and areas of daily use [100% minimum convex polygons]) and three behavior metrics (kill site, bed site, and slow localized movement behaviors in pumas). We hypothesized that: i) movement data will indicate signs of disease onset, such as smaller daily areas of use (minimum convex polygons) and/or less daily distance traveled while clinically infected compared to the disease-free stage of the individuals and ii) the disease stage of the individuals will differ in time length depending on the pathogen (shorter in HPAI vs. longer in *Leptospira* spp).

## Results

**Figure 1:**
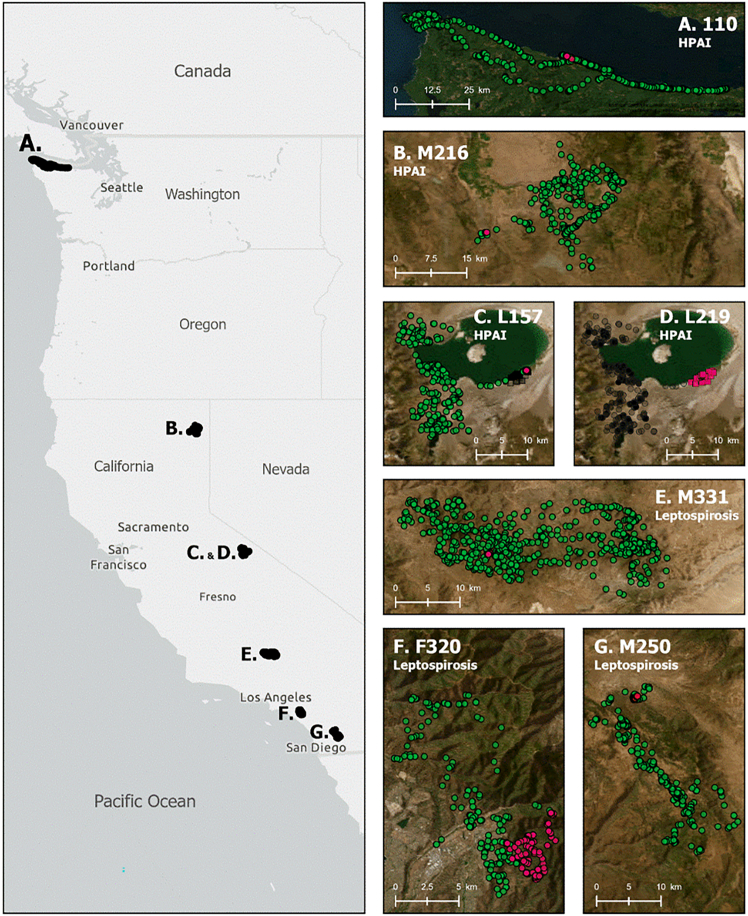
Puma study areas Location of puma research study areas (A–G) in California (Northeastern, Eastern Sierra Nevada, Central, and Southern California) and Washington (left panel) and zoomed-in maps of each highly pathogenic avian influenza (HPAI) and leptospirosis-positive mountain lion movement data with pre (green) and post-infection (pink) locations (right panels A–G).

Over the course of the study period, we identified seven satellite-collared pumas that were determined to have died from infectious disease in California and Washington (USA, Figure 1). Four individuals—two females (mom and daughter, L157 and L219) and two males (M216, “Zeppelin” or 110) died from HPAI. Two males (M250 and M331) and one female (F320) died from leptospirosis.

### Disease onset, movement metrics, and behavior durations in infected pumas

**Figure 2:**
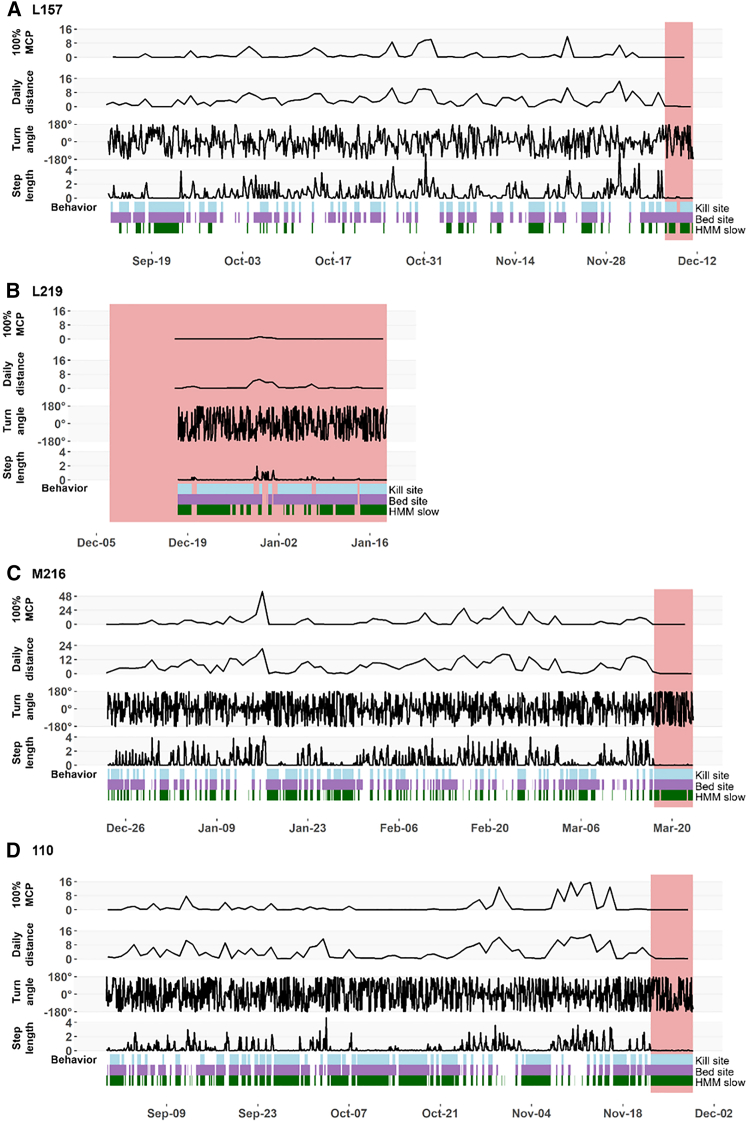
Movement data and behavior durations for pumas with highly pathogenic avian influenza virus infection (HPAI H5N1) in California and Washington, USA (A) L157. (B) L219. (C) M216. (D) 110. The estimated “post onset” period before death is highlighted in pink. L219 shows little movement while collared. Her post onset period is shown starting at the same time as her mother, L157.

**Figure 3:**
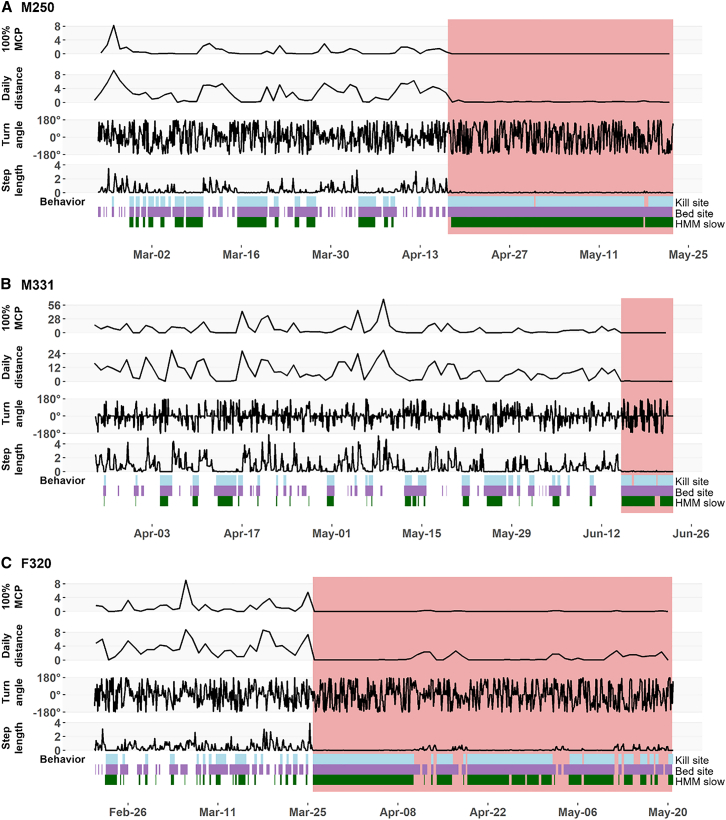
Movement data and behavior durations for pumas with Leptospirosis in California, USA (A) M250. (B) M331. (C) M320. The estimated “post onset” period before death is highlighted in pink.

For six pumas, we were able to identify a “disease onset” point associated with a change in behaviors (Tables 1 and 2; Figures 2 and 3). These six pumas demonstrated a notable decrease in step length after the time of disease onset (Tables 1 and 2). Four of the six pumas (M331, M250, M216, and 110) also showed a significant increase in turn angle absolute value, with the other two pumas (L157 and F320) showing an increase that approached significance. Two pumas (M250 and F320), both of whom died of leptospirosis showed a significant decrease in daily distance traveled, and one puma (F320) showed a decrease in the size of her daily area of use (100% MCP). We could not identify a time of disease (HPAI) onset for one puma (L219), who showed little movement for the short time (33 days) she was collared (Figure 2).

**Table 1:** Interrupted time series model results for movement behaviors (turn angle absolute value (|x|), step length, daily distance moved (km), and area of daily use (100% MCP) of pumas before and after estimated onset of highly pathogenic avian influenza virus infection (HPAI H5N1) in California and Washington, USA

	L157				M216				110			
	Est.	SE	t-value	*p*-value	Est.	SE	t-value	*p*-value	Est.	SE	t-value	*p*-value
**Turn angle |x|**												

Intercept	79.2	5	15.8	<0.001	84.6	3.7	23.1	<0.001	101.2	3.7	27.5	<0.001
Time	0	0	−1.4	0.17	0	0	−1.1	0.282	0	0	−2.3	0.019
Onset	**40.9**	**24.1**	**1.7**	**0.09**	**67.3**	**14.5**	**4.6**	**<0.001**	**29.2**	**14**	**2.1**	**0.037**
Time since onset	0.5	1.6	0.3	0.758	−0.5	0.3	−1.5	0.146	0	0.3	−0.1	0.95

**Step length**												

Intercept	0.4	0.1	5.3	<0.001	0.5	0.1	8.9	<0.001	0.2	0	5.5	<0.001
Time	0	0	2.9	0.004	0	0	1.7	0.094	0	0	4	<0.001
Onset	**−0.8**	**0.4**	**−2**	**0.049**	**−0.6**	**0.2**	**−2.9**	**0.004**	**−0.4**	**0.1**	**−3**	**0.003**
Time since onset	0	0	−0.1	0.914	0	0	0	0.964	0	0	−0.2	0.881

**Daily distance**												

Intercept	2.6	0.6	4.1	<0.001	5.9	1	5.7	<0.001	2.4	0.8	3.2	0.002
Time	0	0	2.2	0.033	0	0	1	0.306	0	0	2.4	0.018
Onset	−4.5	3.6	−1.2	0.218	−7.4	4.6	−1.6	0.111	−5.3	3.4	−1.5	0.127
Time since onset	−0.1	1.3	−0.1	0.936	−0.1	1.1	−0.1	0.959	0	0.9	−0.1	0.955

**100% MCP**												

Intercept	1.1	0.5	2.1	0.037	4	1.8	2.2	0.03	−0.1	0.8	−0.1	0.895
Time	0	0	0.6	0.534	0	0	1	0.326	0	0	3.1	0.003
Onset	−1.7	3.8	−0.4	0.659	−7	8.9	−0.8	0.435	−3.9	3.3	−1.2	0.246
Time since onset	0	1.8	0	0.997	0	2.6	0	0.989	0	0.8	−0.1	0.954

Changes after disease onset that are significant (*p* < 0.05) or approaching significance (*p* < 0.10) are bolded. “Time” describes how the movement metric (i.e., the response variable) changed over time prior to disease onset, “Onset” describes how the movement metric changed at the identified disease onset point, and “Time since onset” describes how the movement metric changed over time after disease onset.

**Table 2:** Interrupted time series model results for movement behaviors (turn angle absolute value (|x|), step length, daily distance moved (km), and area of daily use (100% MCP) of pumas before and after estimated onset of Leptospirosis in California, USA

	M250				M331				F320			
	Est.	SE	t-value	*p*-value	Est.	SE	t-value	*p*-value	Est.	SE	t-value	*p*-value
**Turn angle |x|**												

Intercept	93.7	5.2	17.9	<0.001	44.9	3.4	13.2	<0.001	85.8	7.3	11.7	<0.001
Time	0	0	−1.7	0.099	0	0	−0.3	0.739	0	0	−1	0.313
Onset	**38.8**	**8.4**	**4.6**	**<0.001**	**45.1**	**11.5**	**3.9**	**<0.001**	**15.9**	**9.3**	**1.7**	**0.087**
Time since onset	0	0	−0.7	0.5	−0.5	0.2	−2.5	0.012	0.1	0.1	1.2	0.223

**Step length**												

Intercept	0.4	0	8.4	<0.001	1	0.1	16.7	<0.001	0.4	0	8.2	<0.001
Time	0	0	0.1	0.896	0	0	−4.9	<0.001	0	0	1.5	0.124
Onset	**−0.3**	**0.1**	**−4.8**	**<0.001**	**−0.5**	**0.2**	**−2.3**	**0.022**	**−0.5**	**0.1**	**−8.3**	**<0.001**
Time since onset	0	0	−0.2	0.879	0	0	0.1	0.946	0	0	−0.6	0.563

**Daily distance**												

Intercept	2.7	0.4	6.1	<0.001	11.6	1.4	8	<0.001	2.8	0.5	5.6	<0.001
Time	0	0	0.3	0.764	−0.1	0	−2.4	0.02	0	0	1.8	0.068
Onset	**−2.7**	**0.7**	**−3.7**	**<0.001**	−5.2	5.4	−1	0.335	**−4.3**	**0.7**	**−6.6**	**<0.001**
Time since onset	0	0	−0.2	0.88	0	1	0	0.989	0	0	−1.1	0.295

**100% MCP**												

Intercept	1.2	0.3	4.3	<0.001	11.4	2.4	4.7	<0.001	1	0.4	2.7	0.008
Time	0	0	−1.4	0.159	−0.1	0.1	−1.6	0.105	0	0	1.3	0.195
Onset	−0.5	0.5	−1.1	0.261	−4.6	9.6	−0.5	0.635	**−1.8**	**0.5**	**−3.8**	**<0.001**
Time since onset	0	0	0.6	0.523	0.1	2.1	0	0.968	0	0	−1.1	0.259

Changes after disease onset that are significant (*p* < 0.05) or approaching significance (*p* < 0.10) are bolded. “Time” describes how the movement metric (i.e., the response variable) changed over time prior to disease onset, “Onset” describes how the movement metric changed at the identified disease onset point, and “Time since onset” describes how the movement metric changed over time after disease onset.

Time of disease onset was followed by faster death in individuals infected with HPAI (4, 6, and 7 days) than *Leptospira* (8, 35, and 55 days).

### Behavior durations in infected pumas

**Figure 4:**
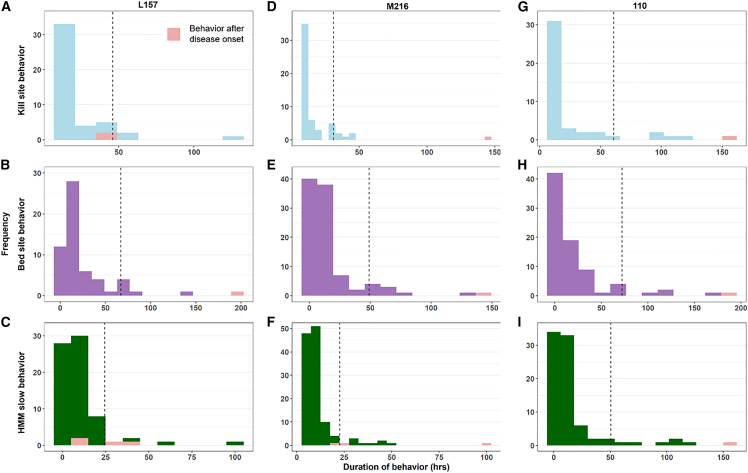
Histograms of the durations of behavior bout for pumas with highly pathogenic avian influenza virus infection (HPAI H5N1) in California and Washington, USA Behaviors occurring after estimated disease onset are highlighted in pink. The dashed lines represent the 95 percentiles of bout length prior to disease onset. No histogram is shown for L219, for which we did not detect normal behaviors. (A–C): kill site, bed site, and slow behaviors for puma L157; (D–F): kill site, bed site, and slow behaviors for puma M216; (G–I): kill site, bed site, and slow behaviors for puma 110.

**Figure 5:**
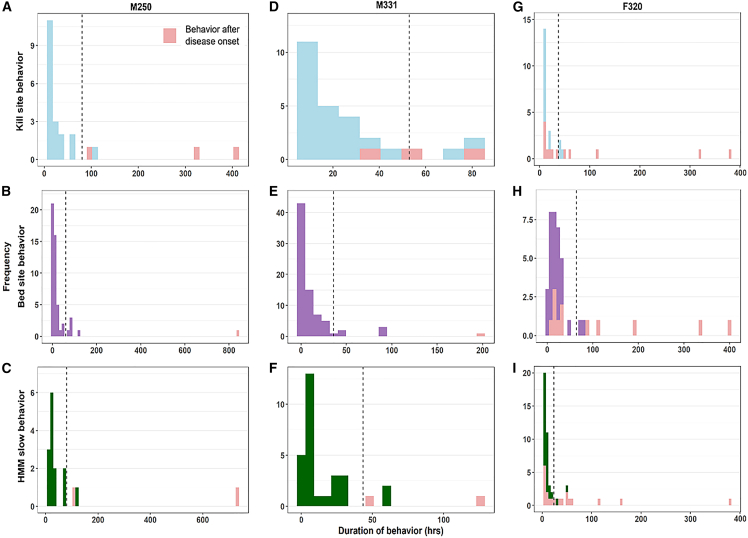
Histograms of the durations of behavior bout for pumas with Leptospirosis in California, USA Behaviors occurring after estimated disease onset are highlighted in pink. The dashed lines represent the 95th percentile of bout length prior to disease onset. (A–C): kill site, bed site, and slow behaviors for puma M250; (D–F): kill site, bed site, and slow behaviors for puma M331; (G–I): kill site, bed site, and slow behaviors for puma F320.

For the six pumas that we identified a time of disease onset for, the duration of apparent kill site behaviors fell outside the 95th percentile for many (M331, F320) or all (110, M250, M216, and L157) bouts post disease onset, indicating that these tended to last longer than pre-onset bouts (Figures 4 and 5). For bed site behavior, post-disease-onset bouts fell well beyond the 95th percentile for five pumas (all except F320). For slow, localized movement behavior from HMMs, the post-onset bouts were all (110, M331, M250) or mostly (M216, L157) beyond the 95th percentile, except for F320. For all three behavioral metrics, F320 additionally exhibited many bouts that were relatively short and thus unusual compared to the other pumas. Nonetheless, this individual’s longest bouts occurred post-disease-onset, and the longest bouts for each behavior occurred immediately post-disease-onset.

## Discussion

This study shed light on how infectious disease is associated with variation in selected movement and behavioral metrics in a large field, with infected individuals showing altered movement. We demonstrated that telemetry data obtained from satellite-GPS collars combined with pathologic determinations can help inform disease onset and post-infection behavior in wildlife species.^48^ Although movement may offer a more direct medium for disease surveillance, the use of movement data to observe infection-induced behavioral shifts is still underexplored.^49^^,^^50^ The ability to identify infected individuals from movement behavior may be of value when diagnosis is difficult or invasive and may also help researchers infer the approximate onset time of clinical signs.^48^^,^^49^

A recent comprehensive framework emphasizes integrating historical and near-real-time biologging data with disease surveillance to provide insights into infection-induced behavioral changes.^51^Our study may fit into this framework, as we used a species that could serve as a potential sentinel host species due to its relevance to susceptibility to infectious diseases, disease-symptomatic behavior, movement characteristics, and suitability for biologging.^51^ Pumas have already been proposed as useful sentinel species for another zoonotic infectious disease (plague).^52^ Metrics included in our research may also identify deviations from baseline behaviors that could be used as an early alert of a disease outbreak or for the detection of a disease occurrence, both especially relevant for zoonotic diseases.^51^

Movement data have been used to identify potential anthrax diagnoses in cheetahs (*Acinonyx jubatus*), where satellite telemetry data aided in reconstructing the behavior of deceased individuals. The investigation of movement data led researchers to identify the consumption of an anthrax-positive zebra (*Equus zebra),* which caused the lethal infection in the cheetahs.^48^ Prior to Portas et al.,^48^ the examination of the effects of clinical disease on the movement behavior of solitary wild felids had been unexplored. However, such research had been examined in African lions (*Panthera leo*), following an outbreak of canine distemper virus (CDV) in 1994 that led to the reduction of territory size and overlap of lion prides.^53^ Similar work has also been conducted on canids (i.e., wolves and coyotes) suffering from sarcoptic mange.^20^^,^^21^^,^^54^

We were able to identify the disease onset in six pumas based on observed behavioral shifts. This disease onset followed a faster death in individuals infected with HPAI than *Leptospira*, which is consistent with what is described in the literature for domestic cats.^45^ We were unable to determine a disease onset date for one individual infected with HPAI (L219), due to the limited movement this puma exhibited for the entirety of the short period of time she was collared. This female puma was a subadult and mother-dependent at the time of her death. Although this subadult puma’s reduced movement could be a potential sign of sickness behavior from the moment she was collared, young pumas that are still dependent on their mother may move less than subadult individuals dispersing from the natal range. Her movement pattern may be further exaggerated by the decreased movements of her diseased mother. Nevertheless, we acknowledge that inferring behavioral shifts from mother-dependent individuals could be challenging due to the limited extent of movement patterns at this age.

Despite the more acute and fulminant outcome observed in pumas infected with HPAI (i.e., shorter period between clinical disease onset and death), all six adult pumas studied showed changes in at least one movement metric. Step length was the most consistent metric showing clinical signalment, independently of the infection type. We also observed a significant decrease in daily distance for two of the three individuals infected with *Leptospira*, but none of the individuals with HPAI. Decreases in movement speed (i.e., the inverse of step length) have been recorded in chronic disease-infected deer^19^ and other carnivorans with chronic infectious diseases. Cross et al.^21^ found that wolves with sarcoptic mange showed a decrease in daily distance with late-stage infection, reducing total distance moved more than earlier stages. Raccoon dogs (*Nyctereutes procyonoides*) and wombats (*Vombatus ursinus*) with sarcoptic mange also showed a decline in daily distance traveled, with the study authors proposing that the altered movements may be an attempt to reduce energy expenditure associated with movements as a trade-off against fighting infection.^55^^,^^56^ While daily travel distance is a widely used measure of overall movement activity, and in our study it clearly captured reduced locomotor effort during infection, it fails to reflect how far individuals move from a starting or central location. In contrast, net daily displacement (or net squared displacement) offers a complementary lens by illustrating the degree to which movement is spatially directed or confined—highlighting whether individuals are range-restricted or still exploring despite illness. Prior methodological frameworks have successfully combined total movement metrics (such as daily distance) with net displacement and trajectory tortuosity (e.g., the ratio of displacement to distance—i.e., straightness index) to characterize movement strategies more richly, particularly in central place foraging contexts.^57^Applying both metrics in tandem may thus enhance the detection of disease-related behavioral shifts in movement strategy.

Turn angle increased for four individuals (110, M331, M250, and M216) and approached significance for the other two. This differs from what has been observed in CWD-infected versus negative control deer (*Odocoileus* sp.) and in a red fox (*Vulpes vulpes*) population before and after a mange outbreak, where turn angle did not differ statistically between pre- and post-disease onset.^19^^,^^58^ In the red fox population, this lack of turning angle significance could indicate an inbuilt species- or habitat-specific search strategy.^58^ In our case, the differences observed in turn angle may indicate an increase in searching or inability to move intentionally while in the clinical course of the infection. While paired with shorter step lengths, this increase in turn angle may typify movement that is more random (e.g., as observed in altered movement behavior at habitat edges^59^ or locally confined (as often captured by HMMs).^60^ Due to the coarser resolution of our data, results from our turning angles should be interpreted with caution, since this metric is most informative at fine temporal scales. At our temporal resolution, such processes are better captured with metrics such as daily net displacement—or its squared equivalent—which provides a more robust metric for discerning broad-scale ranging versus central place-oriented behavior, as differences in return-to-origin distance better reflect shifts in movement domain.^61^

One *Leptospira* infected individual (F320) demonstrated a significant decrease in the daily amount of space used, measured as daily 100% minimum convex polygons. Changes in space use/territoriality followed by an infection have been reported in other species (e.g., coyotes, wombats, raccoon dogs, African lions, and Tasmanian devils).^20^^,^^53^^,^^55^^,^^56^^,^^62^ Based on the lack of significance for this metric for all but one infected puma, we hypothesized that significant changes in the amount of space used could be easier to identify in chronic/debilitating diseases. In our case, this individual presented changes in her movement for almost two months after the disease onset, while the other individuals presented a more rapid fatal outcome that led to fewer relocations. Although this metric is conceptually simple, it is still valuable to describe third-order resource selection,^63^ which could be altered during clinical stages.

In contrast to this coarse metric, higher resolution data have proven to be more useful in identifying subtle changes in movement behavior. In this regard, a bottom-up approach to movement analysis can serve to simulate animal movement in diverse contexts, such as identifying when individuals are sick.^64^

In addition to movement metrics, behavior metrics also changed after disease onset. Pumas tend to show reduced movements at kill sites, where they stay in the vicinity to feed on carcasses, and at bed sites, where they rest.^65^^,^^66^^,^^67^ Movement patterns can be used to estimate when pumas were exhibiting specific behaviors, although on-the-ground confirmation is needed to confirm whether movements occurred at kill or bed sites. Here, we applied the movement criteria for bed and rest site behavior to our pumas and showed that the durations of these apparent kill site and bed site behaviors are often longer post-disease onset. While these movement criteria can help identify potential bouts of kill site and bed site behaviors in healthy pumas, unusually long bouts of these “behaviors” can indicate that an animal is unhealthy. It is unclear what behaviors diseased pumas actually exhibit during these bouts of kill site or bed site behavior. For example, longer bouts of apparent kill site behavior after disease onset may indicate that diseased, weak animals may stay longer on a kill, or altered movement patterns may result in resting behavior that looks such as kill site behavior. However, we highlight that one of the pumas (M216) was found dead in a kill site eating two juvenile pumas (both of which also tested positive for HPAI), and behavioral classification did identify him as exhibiting kill site behavior prior to death (Figure 2C).

Hidden Markov models also infer the underlying behavioral states from movement patterns, grouping movements into broad categories such as straight, long distance movements (fast) and tortuous, local movements (slow).^60^^,^^68^ As with the kill site and bed site movements, we found that pumas tended to have longer bouts of slow, local movement after disease onset. Thus, in addition to movement metrics, metrics that infer behaviors from location data also show characteristic changes post infection with *Leptospira* spp. and HPAI. However, we note that these behavioral metrics are typically derived from “raw” movement metrics (e.g., step length), so the simplest approach to identifying diseased movements is to use these movement metrics directly. Nonetheless, using multiple indicators of disease may be most informative since pumas in our study differed in which movement and behavioral metrics showed changes post-disease onset.

This research demonstrated the first effort to examine movement and behavioral metrics in Leptospira and HPAI-infected pumas, which may aid in understanding the clinical significance of both infectious diseases in this species. Future studies should consider rapid declines in certain movement metrics (e.g., step length, movement speed) while tracking pumas, as they may serve as an indicator of overall individual health. This approach may serve as a management and conservation tool for species of conservation significance (i.e., specially protected species or state/federal endangered) and could improve the parameterization of spatially explicit disease transmission models. For certain populations, the identification of drastic behavioral shifts may prompt a timely response to recapture and evaluate individuals for removal from the wild for disease testing, treatment, rehabilitation, and reduction of disease transmission rates.

### Limitations of the study

We acknowledge our inferences may be limited due to the small sample size; however, opportunities to examine the movement behavior of diseased carnivores are rare and warrant attention. We also note that we only used puma data from the three months prior to death in an attempt to limit the extent to which factors such as age or seasonality could underlie changes in movement patterns. Fine-scale (i.e., high frequency) movement data are generally less influenced by seasonality than coarser metrics, since they reflect short-term behavioral modes such as traveling or searching rather than broader seasonal patterns.^69^ However, we posit that, barring any confounding effects, disease-induced changes in movement and behavioral metrics may be even more profound when compared to long-term patterns.

## Resource availability

### Lead contact

Fernando Nájera; fnajera@ucdavis.edu.

### Materials availability

This study did not generate new unique reagents.

### Data and code availability

• Accession codes can be found in the key resources table.
• Data used in this study are located in Zenodo: https://doi.org/10.5281/zenodo.15623339.
• Code used in this study includes the following packages: Behavioral Change Point Analysis Package, Move Package, MoveHMM Package, and Adehabitat HR.

## Acknowledgments

We appreciate the veterinary pathologists at the California Animal Health and Food Safety Laboratory System of the University of California, the California Department of Fish and Wildlife, and the Washington Department of Fish and Wildlife who provided necropsy and pathology services. We thank all the field biologists (especially Juan Gonzalez and Lina Vu) and veterinarians who have been involved during the capture, anesthesia, and GPS-collaring of the study pumas across our study sites in California and Washington. We thank the Administration for Native Americans, the USFW Tribal Wildlife Program, the National Geographic Society, the Fat Cat Feline Fund, the Ayers Wildcat Conservation Trust, Dorothy Bahna, the Stevenson family, Miriam and Gerry Scully, and others. We thank A. Stratton and C. Kupar for field work.

We also thank all of the municipal, county, state, federal, tribal, and private land owners and managers who allowed field activities on their properties across both states. We are grateful for the financial support provided by the CDFW Federal Aid and Wildlife Restoration Grant(s) F22AF00236, F23AF00236, and F24AF00236; the CDFW and Federal Assistance in Wildlife Grants*;* the California Wildlife Program-Wildlife Conservation Network, the San Diego Zoo Wildlife Alliance, and the San Diego Association of Governments. This article is #25-004 of the CDFW Mountain Lion Conservation Program.

## Author contributions

Conceptualization, F.N., data curation , F.N. and S.U.; formal analysis, F.N. and S.U.; funding acquisition, F.N., T.W.V., D.K.G., J.V.L., T.R.S., and L.M.E.; investigation, F.N., T.W.V., D.K.G., J.V.L., T.R.S., L.M.E., J.F.R., P.J., and D.L.C.; methodology, F.N., S.U., T.W.V., D.K.G., J.V.L., T.R.S., L.M.E., and D.L.C.; project administration, F.N., T.W.V., D.K.G., J.V.L., and L.M.E.; resources, F.N., T.W.V., D.K.G., J.V.L., T.R.S., L.M.E., J.F.R., P.J., and D.L.C.; supervision, F.N.; validation, S.U.; visualization, F.N. and S.U.; writing – original draft preparation, F.N., S.U., J.V.L., and D.L.C.; writing – review and editing, F.N., S.U., T.W.V., D.K.G., J.V.L., T.R.S., L.M.E., J.F.R., P.J., and D.L.C.

## Declaration of interests

The authors declare no conflicts of interest regarding this article.

## STAR★Methods

### Key resources table

**Table:** 

REAGENT or RESOURCE	SOURCE	IDENTIFIER
**Deposited data**		

Data	This paper	https://doi.org/10.5281/zenodo.15623339

**Software and algorithms**		

RStudio	R Code Team	https://www.r-project.org
Behavioral Change Point Analysis Package	CRAN	https://cran.r-project.org/package=bcpa
Move Package	CRAN	https://cran.r-project.org/package=move
MoveHMM Package	CRAN	https://cran.r-project.org/package=moveHMM
AdehabitatHR	CRAN	https://cran.r-project.org/package=adehabitatHR

### Experimental model and study participant details

Capture and collaring of pumas took place in four different ecological zones in California and on the Olympic Peninsula in Washington from 2020 to 2023 (Figure 1). Pumas belonged to a wide spectrum of projects aiming to elucidate aspects of puma ecology, including landscape use, landscape connectivity, foraging ecology, and/or interaction with other species. For this study, we collected data from individuals that died as a consequence of leptospirosis or HPAI while being satellite-collared.

In Washington, the study area encompasses the Olympic Peninsula and coastal Washington west of the I-5 corridor. It is bounded by the Pacific Ocean to the west, the Strait of Juan de Fuca to the north, the Puget Sound and I-5 corridor to the east, and the Columbia River to the south. Most of the Olympic Peninsula falls within Olympic National Park (3,733 km^2^) or the Olympic National Forest (2,540 km^2^). The rest consists of vast tracts of public and privately managed forestry lands dotted with small to medium-sized towns. The habitat consists of temperate rainforest, glaciated rugged mountains, and a rocky, broken coastline. Rainfall is variable with peaks of 300–500 cm in the west to as little as 40 cm in the northeastern corner of the peninsula.^70^

In northern California, research occurred on the Modoc Plateau, covering 10,890 km^2^. Temperatures and annual precipitation range from -11°C to 32°C and between 17.8 and 121.9 cm, respectively.^71^^,^^72^ Elevations range between 1219 to 2973 m across the study area. The majority of the study area is public land (US Forest Service, Bureau of Land Management, and U.S. Fish and Wildlife), and the dominant vegetation is juniper (*Juniperus occidentalis*) woodlands, sage steppe, and conifer forest, along with agricultural lands.^72^

In the eastern Sierra Nevada (i.e., the eastern escarpment of that range and valleys immediately to the east), elevations ranges from 1,060 to 4,421 m. Low elevations (1525–2500 m) are characterized by sagebrush-steppe (*Artemesia tridentata*) vegetation; mid elevations (2500–3300 m) by piñon (*Pinus monophyla*), Sierra juniper (*P. grandis*) woodland, subalpine meadows, and forest; and high elevations (> 3300 m) by sparse alpine vegetation interspersed with meadows.^73^ Snowfall is common at elevations above 1800 m and the higher elevations receive 5000–15000 mm of snow annually.^74^ In contrast, annual precipitation is as low as 150 mm at lower elevations in the rain shadow of the Sierra Nevada. Temperatures range greatly over the year at different elevations (low: -2–32°C; high: -14–15°C).^74^

The central California study area included the Tehachapi Mountains (approximately 2,100 km^2^) which connect the southern end of the Sierra Nevada with the coast ranges of California.^75^ The elevation ranges from 368 to 2360 m.^76^ The main vegetation type is oak woodlands, including canyon live oak (*Quercus chrysolepis*), interior live oak (*Q*. *wislizeni*), blue oak (*Q*. *douglasii*), California black oak (*Q*.*s kelloggii*), scrub oak (*Q*. *ilicifolia*) along with ponderosa pine (*P*. *ponderosa*) and gray pine (*P*. *sabiniana*).^77^ Mean precipitation ranges from 0-250 mm, with higher elevations resulting in snowfall.

The study area in Southern California included the Santa Ana Mountains (2,060 km^2^; 0-1,717 m elevation) and the eastern Peninsular Ranges (14,520 km^2^; 0-3200 m elevation). In the Santa Ana mountains, vegetation communities include chaparral, oak woodlands (*Q*. *engelmannii* and *Q. agrifolia*), coastal scrub, annual grasses, and coniferous forests at higher elevations, along citrus, avocado orchads and other non-native vegetation.^78^ Eastern Peninsular vegetation communities include a mosaic of chaparral, sage scrub (*Artemisia californica*, *Adenostoma fasciculatum, Larrea tridentata*, *Ambrosia dumosa*), oak woodlands (*Q*. *dumosa, Q. agrifolia, and Q. engelmannii*) open-conifer forests (*P*. *ponderosa, P. jeffreyi, and P. coulteri*), and grasslands.^79^ Temperatures reach 24°C and annual precipitation ranges from 500-700 mm (Santa Ana Mts) and 250-900 mm (Eastern Peninsular Range).^78^^,^^80^

### Method details

#### Puma capture and disease testing

We captured pumas using trained scent-hounds^81^^,^^82^ and cage traps baited with road-killed deer. Cage traps were placed at bait sites where a puma had scavenged on road-killed deer set by the field team. Cage traps were continuously monitored after they were set via trap alarm system.^75^ Anesthetic combinations included tiletamine-zolazepam and medetomidine-ketamine in California^82^ or butorphanol-azaperone-medetomidine (BAM; 1.5 cc/45 kg of BAM [per ml, 27.3 mg Butorphanol Tartrate, 9.1 mg Azaperone Tartrate, and 10.9 mg Medetomidine HCL]) in Washington. During animal processing, some studies placed a numerical tag in one ear and a tattoo in the opposite ear, and we collected biological samples (blood, hair, and whiskers) from each individual. We fit pumas with GPS collars (Vectronics Aerospace, Lotek Wireless) programmed at one to four-hour fix rates, which were dependent on study objectives. We monitored the body temperature, oxygen saturation, respiration and heart rate of anesthetized pumas every 5 min and anesthesia was reversed with an equal dosage of atipamezole (0.2 mg/kg, for medetomidine-ketamine) or atipamezole (0.2 mg/kg) + 0.5 ml naltrexone (for BAM). Deaths of radio-collared pumas were detected by a remote satellite mortality signal from the GPS collar, and we determined the cause of death by field investigation and a standard postmortem examination. We operated under multiple state and institutional capture and research protocols including: (1) Institutional Animal Care and Use Committees (University of California, Davis protocol #22408 and University of Idaho protocols IACUC-2020-15 and IACUC-2017-79); (2) Memoranda of Understanding and Scientific Collecting Permits from the California Department of Fish and Wildlife; (3) California Department of Fish and Wildlife Capture Plan.

We diagnosed leptospirosis in California pumas via serology, PCR testing, histology and immunohistochemistry along with pathological manifestations of the disease at California Animal Health and Food Safety Laboratory (CAHFS, Davis, California, USA).^31^ For the serological testing, we used the microscopic agglutination test (MAT)^83^ to detect antibodies against six serovars of *Leptospira* spp.: *L. interrogans* serovars Pomona (serogroup Pomona), Hardjo type Prajitno (serogroup Sejroe), Canicola (serogroup Canicola), Copenhageni (serogroup Icterohaemorrhagiae), and Bratislava (serogroup Australis); and *L. kirschneri* serovar Grippotyphosa (serogroup Grippotyphosa), chosen to be consistent with the local CAHFS standard protocols.^31^ For histological evaluation and immunohistochemistry (IHC), at necropsy, we collected samples of kidneys into 10% neutral-buffered formalin and then embedded in paraffin. After being stained with H&E and evaluated microscopically, we subjected kidney samples with evidence of interstitial nephritis to Steiner staining and IHC using Leptospira multivalent fluorescent antibody conjugate (National Veterinary Services Laboratory LEP-FAC, CAHFS.^31^ For pumas with histological lesions consistent with HPAI infection (e.g., necrotizing encephalitis) at the necropsy performed by CAHFS (California) and the Washington Animal Disease Diagnostic Laboratory (WADDL, Washington), the National Veterinary Services Laboratories (NVSL) in Ames, Iowa^46^ confirmed diagnosis of HPAI H5N1 infection in pumas by testing brain tissues by performing sequential real-time reverse transcription PCR (rRT-PCR) for influenza A viruses (IAV) and the H5 2.3.4.4 virus subtype.

### Quantification and statistical analysis

We selected a three-month period because we expected pumas to be disease-free during some proportion of this time based on movement patterns and the pathological findings of the necropsies (i.e., none of the pumas would have been able to survive more than eight weeks based on the severity of the lesions found in the postmortem examination). We first used the *interpolateTime* function in the *move* package in R to interpolate missing data points.^84^ We did this to ensure we had either a real or interpolated location every four hours (one individual), three hours (two individuals), or two hours (four individuals), based on the programming of the collar. We then calculated step lengths (i.e., the distance between consecutive GPS locations),^85^ turn angles (i.e., the difference in direction for two successive moves),^85^ and daily travel distances (i.e., step lengths summed for each 24 hr period). We also calculated daily 100% minimum convex polygons as a measure of the area of space used each day. In addition to these four-movement metrics, we considered three behavioral metrics that are associated with reduced movement, and which could appear similar to diseased movements: kill site visits, bed site visits, and slow or resting movement according to a hidden Markov model (HMM). For each GPS point, we identified which of these three behavior(s) the puma appeared to be exhibiting. We performed these analyses using the *move*, *moveHMM, and adehabitatHR* packages in R 4.3.2.

We considered pumas to be exhibiting “kill site behavior” when there were at least 5 consecutive locations within 150m of each other, with at least one location occurring at night.^65^^,^^66^ We considered pumas to be exhibiting “bed site behavior” when there were at least two locations within 150m of each other, that occurred at least 4h apart, but within 2 weeks.^67^ Lastly, we fit HMMs to the data to estimate two behavioral states (slow/resting and fast/traveling) with different distributions of step lengths and turn angles.^68^^,^^86^ We modeled step lengths using a gamma distribution and turn angles using von Mises distribution. Based on the empirical distributions of the step lengths, we tried 20 sets of randomly selected initial step length parameter values ranging from 10–800 and 50–2000 (mean step length) and 5–500 and 20–1000 (step length standard deviation).^68^^,^^86^^,^^87^ For the initial turn angle parameters, we used π and 0. We selected the best-fitting model using log-likelihoods and then used the Viterbi algorithm to identify which GPS locations were associated with the “slow” state. We identified “bouts” of behavior as consecutive GPS fixes which met the requirements for each behavior, with the number of GPS fixes indicating the duration of the bout.

For each puma, we used movement and behavior metrics to visually determine when activity began to appear abnormal prior to death, which we called “disease onset”. We then used behavioral change point analysis (BCPA), via the package *bcpa*^88^ to statistically confirm the identified disease onset time. In all cases, BCPA identified a change point within hours of the estimated disease onset (mean: 2.8 hrs, range: 0-6 hrs). We then used interrupted time series model to test whether disease onset was associated with a change in each of the three movement metrics. For turn angle, we used the absolute value for this analysis. For each behavior metric, we used only data prior to the onset of abnormal activity to calculate bout durations. Using log-normal distribution, we examined whether post-onset bouts of each behavior last longer than pre-onset bouts by determining whether post-onset behavioral bouts fell outside of the 95^th^ percentile of pre-onset behavioral bouts.
